# Non-communicable diseases and global health governance: enhancing global processes to improve health development

**DOI:** 10.1186/1744-8603-3-2

**Published:** 2007-05-22

**Authors:** Roger S Magnusson

**Affiliations:** 1 University of Sydney, 173-175 Phillip St, Sydney, NSW 2000, Australia

## Abstract

This paper assesses progress in the development of a global framework for responding to non-communicable diseases, as reflected in the policies and initiatives of the World Health Organization (WHO), World Bank and the UN: the institutions most capable of shaping a coherent global policy. Responding to the global burden of chronic disease requires a strategic assessment of the *global processes *that are likely to be most effective in generating commitment to policy change at country level, and in influencing industry behaviour. WHO has adopted a legal process with tobacco (the *WHO Framework Convention on Tobacco Control*), but a non-legal, advocacy-based approach with diet and physical activity (the *Global Strategy on Diet, Physical Activity and Health*).

The paper assesses the merits of the Millennium Development Goals (MDGs) and the FCTC as distinct global processes for advancing health development, before considering what lessons might be learned for enhancing the implementation of the Global Strategy on Diet. While global partnerships, economic incentives, and international legal instruments could each contribute to a more effective global response to chronic diseases, the paper makes a special case for the development of international legal standards in select areas of diet and nutrition, as a strategy for ensuring that the health of future generations does not become dependent on corporate charity and voluntary commitments. A broader frame of reference for lifestyle-related chronic diseases is needed: one that draws together WHO's work in tobacco, nutrition and physical activity, and that envisages selective use of international legal obligations, non-binding recommendations, advocacy and policy advice as tools of choice for promoting different elements of the strategy.

## Background

Since 1970, life expectancy at birth has improved steadily, rising 7, 8 and 9 years, respectively, within high, middle and low income countries to reach 79, 70 and 58 years, as measured from data for the period 2000–2005 [1]. While the underlying causes of these gains continue to be debated [2], longer life expectancy has resulted in the global predominance of non-communicable diseases as both the leading cause of death, and of disease burden. According to World Health Organisation (WHO) estimates, non-communicable diseases accounted for nearly 59% of the 57 million people who died in 2002 [3]. In the same year, non-communicable diseases also outstripped both communicable diseases, and injuries, as the leading cause of chronic illness worldwide, accounting for nearly 47% of the 1.49 billion years of healthy life "lost" to illness, as measured in DALYs [3].

Within developing countries, this "epidemiological transition" reflects the higher proportion of adults in the population (due to declines in both fertility rates and infant mortality) who, over time, age and become ill from diseases that disproportionately affect adults [4]. In addition, it reflects the rapid rise in behavioural risk factors including smoking and high-sugar, high-fat diets. The "nutrition transition" towards diets that are richer in saturated fats and poorer in complex carbohydrates and dietary fibre, fruit and vegetables; the growth of urban lifestyles involving less physical exertion; and the promotion and rising consumption of tobacco and alcohol, have set the scene for "lifestyle epidemics" to become the greatest health challenge of the twenty-first century [5-9].

While the proximate behavioural risk factors for non-communicable diseases are well-known, the underlying environmental causes are both complex and global in scale [10,11]. Environmental factors underlying the nutrition transition include the industrialization of food production, the growth of sophisticated supply chain management on a global scale, the expansion of market economies in developing countries, the growing concentration of global food manufacturers as a result of mergers and acquisitions, and the rapid growth of supermarkets in the developing world. Rising incomes, price differentials favouring the cheap production of energy-dense foods, growing urbanization and rapid growth in demand for pre-prepared foods, are also key factors [12-16]. While "no food manufacturer commands a substantial share of total world processed food sales", focused growth has nevertheless created "concentrated markets...at specific product and country levels" [17]. In 2002, over seventy-seven percent of global food sales were of processed foods and beverages [18]. To that extent, processed food manufacturers exercise a significant influence over global nutrition. Market concentration is even more evident in the tobacco market, where global cigarette production is dominated by a small number of British, American and Japanese corporations which have benefited from trade liberalization and are pursuing growth in developing countries [19].

This paper assesses progress in the development of a global response to non-communicable diseases, as evidenced by initiatives and policies of the World Health Organisation (WHO), World Bank and the UN: the institutions most capable of shaping a coherent global policy. Responding to the global burden of chronic disease requires a strategic assessment of the *global processes *that are likely to be most effective in encouraging the implementation of effective policies at country level, and in influencing industry behaviour. Possible processes for driving policy change, as illustrated in different global initiatives, include: (i) international legal instruments creating legal obligations on signatories to implement certain policies; (ii) economic incentives; and (iii) partnerships between global and national stakeholders for the advancement of shared policy objectives.

A feature of both diet and tobacco-related diseases is the presence of powerful multinational corporations and the challenge of regulating their products. WHO has adopted a treaty-based approach with tobacco [20], but a facilitative, advocacy-based approach for diet and physical activity [21]. While the rapid entry into force of the *WHO Framework Convention on Tobacco Control *(FCTC) has focused global attention around this problem and enhanced WHO's standing, the challenge of implementing the FCTC and developing effective partnerships to resist the influence of the tobacco industry at the country level, is ongoing. WHO's *Global Strategy on Diet, Physical Activity and Health *(GSDPAH), anticipates a broad coalition of agencies and stakeholders working with countries and the food industry towards implementation, but to date, progress has been patchy [22,23].

This paper argues that global and national partnerships, economic incentives, and international legal instruments could each contribute to a more effective global response to chronic diseases. While the FCTC may not be the appropriate model for diet and obesity, the paper makes a special case for the development of binding international standards in select areas of diet and nutrition. At present, the conceptual framework for global action on "lifestyle-related" chronic diseases is largely embodied in two WHO initiatives: the FCTC and GSDPAH. A broader frame of reference is needed: one that links together WHO's work in tobacco, nutrition and physical activity, and even alcohol, and that envisages the strategic use of international legal standards, non-binding international recommendations, advocacy and policy advice as tools of choice for promoting different elements of the strategy.

## The impact of non-communicable diseases in developing countries

An impressive body of evidence supports the case for urgent action in response to the growing burden of chronic disease in developing countries [15,24-29]. The epidemiological transition from communicable to non-communicable diseases is far from uniform or complete, especially in sub-Saharan Africa. Over the period 1990–2001, the share of global deaths from HIV/AIDS grew from 2% to 14% [25], reducing life expectancy at birth to less than 40 years in several sub-Saharan African countries [3]. Mortality in children less than 5 years has declined in all regions since 1990, yet it still accounted for nearly 20% of all deaths in 2001 [25]. Of these 10.5 million deaths, nearly all were in low and middle income countries: diarrhoeal diseases, lower respiratory tract infections and malaria were among the leading causes [3].

Heart disease, stroke, and chronic obstructive pulmonary disease are now adding a "double burden" on low and middle income countries, with epidemics of diabetes and lung cancer reflecting rising rates of smoking and obesity. In numerical terms, heart disease kills 17 million people per year, in comparison to three million deaths from AIDS [26]. In 2001, heart disease and stroke were the leading cause of death in both high income, and low-middle income countries, accounting for 27% and 21%, respectively, of total deaths in each group [25]. However, due to their larger populations, nearly 83% of these deaths occurred in developing countries [25]. Evidence suggests that illness and death from cardiovascular disease in developing countries occurs at a younger age, cutting into the productive years and undermining the benefits of the lower dependency rate enjoyed by developing countries with younger populations [3,26]. With more than 1.1 billion adults overweight worldwide, and 312 million of them obese [30], diabetes is expected to double from 171 million to 366 million cases over the period 2000–2030. Developing countries will have far higher numbers overall (especially India, China, Southeast Asia and the Western Pacific), with incidence peaking in the 45–64 age group [31]. Overall, some 80% of DALYs from chronic diseases fall on populations less than 60 years of age [28].

In 2000, tobacco caused an estimated 4.83 million premature deaths, 12% of total global mortality in those 30 years and above [32]. Half of these deaths were in the developing world. More than 1 billion of the world's 1.3 billion smokers living in developing countries; as a result, the future epidemic of tobacco-related diseases will impact overwhelmingly on the developing world [33]. While total cigarette consumption has been decreasingly sharply in the developed world since 1975, it continues to rise in developing countries, largely due to population growth [33]. Even assuming that tobacco control efforts are successful in reducing smoking prevalence by 1% each year in every country from 2003, there will still be over 1.3 billion smokers in 2010, and 1.45 billion in 2025 [33]. Overall, between one-half and two-thirds of long-term smokers will die from tobacco-related diseases [34]. Peto and Lopez estimate that if current patterns persist, tobacco will kill about ten million people per year by 2030 (by which time seven out of ten deaths will occur in developing countries), with 150 million deaths to 2025, and up to 300 million from 2025–2050 [35].

## Where do global processes fit within global health policy?

Lee, Fustukian and Buse provide a helpful framework for disentangling four dimensions of global health policy-making [36]. The first component is the *policy actors*: who exercises sufficient (political) power to drive policy and to influence decision-making at the global level? In the area of non-communicable diseases, the United Nations, WHO, the Food and Agricultural Organisation (FAO), the World Bank, the World Trade Organisation (WTO), and the Codex Alimentarius Commission, deserve particular attention. Secondly, what are the *processes *through which policy is developed, and implemented? What kinds of interactions and relationships between policy actors are most effective in leading to global health improvements?

The third component is the *context *of global policy development. The pervasive theme here is globalisation itself: a process reflected in the disappearance of boundaries, the increasing integration of the global economy, and flowing from that, the intensification of transnational interactions and influences across physical, political, social and cultural borders [37]. The point is often made that globalization has diminished the capacity of countries to deal effectively with major health threats occurring within their borders, creating new imperatives for international cooperation, and thrusting new responsibilities onto global actors, civil society and the private sector [38-40]. Globalization creates, in other words, new *process challenges *to an effective response to national health problems. Coordinated strategies involving partnerships between international agencies, the private sector and nongovernmental organizations (NGOs) are required because few policy actors – global or not – are capable of single-handedly driving policies across multiple sectors at country level in order to lay the groundwork for population-wide health improvements.

The specific things that policy actors seek to achieve through these global activities and collaborations comprise the fourth component: the *content *of global health policy. With non-communicable diseases, there is broad recognition that effective strategies should address universal prevention at the population level, selective (or primary) prevention directed at high-risk groups, and targeted (or secondary) prevention, and treatment, for those with existing conditions [10]. Typically, policy prescriptions for prevention direct attention to the key determinants for the diseases in question and to the priority settings for intervention. These determinants include global factors and processes (as influenced by global policy actors), socioeconomic factors and aspects of the political, social and physical environment at country level, individual "lifestyles" and behaviours, access to health services, as well as the design and functioning of national health systems. Effective regulation of the structural and environmental determinants of non-communicable diseases requires interventions that extend well beyond the health sector [41]. Policy influence is required in areas including agriculture, finance and taxation, education, recreation and sports, media and communication, transportation, and urban planning [21]. Coordinating mechanisms are needed to facilitate inter-sectoral and interdepartmental cooperation [21].

This paper focuses not on the content of policies for non-communicable diseases, but on the global processes best suited to achieving enduring policy change at country level, and to reducing long-term harm from industry practices. One available process is international law. Multilateral agreements include "hard law" conventions that contain legally-binding obligations, such as the *WHO Framework Convention on Tobacco Control *(FCTC) [20]. They also include "soft law" resolutions and declarations, such as the General Assembly's *United Nations Millennium Declaration *[42], which, although intended to create a good faith obligation on member states to work towards the MDGs, remains a recommendation with normative effect only [43]. Global agencies can also develop guidelines, strategy documents and policy frameworks to assist member countries: WHO's *Global Strategy on Diet, Physical Activity and Health *(GSDPAH) falls into this category [21].

Discussion of the capacity for multilateral processes to advance global health goals raises debate over the extent to which global agencies, such as WHO, merely provide a framework for members to express and pursue their interests, or whether they retain an independent capacity to influence global policy in their own right [44,45]. The development of global norms and strategies is an intensely political process, as the FCTC and GSDPAH illustrate [46,47]. In WHO's case, the better view may be that the secretariat, executive board and World Health Assembly (WHA) each operate in a fluid political environment that provides possibilities for, as well as real-world constraints upon, innovation and leadership. Early resolutions supporting the development of the FCTC, for example, were supported by the WHO executive board and adopted by the WHA, but over the opposition of the secretariat [48]. Little progress was made, however, until former Director-General Gro Harlem Brundtland took office in 1998, making tobacco control a top priority, establishing the Tobacco Free Initiative within her cabinet, and reviving the earlier (1996) mandate to begin multilateral negotiations for a framework convention [49,50].

## What place for non-communicable diseases on the health development agenda?

The process of globalization has dramatically expanded the scale of international cooperation and led to wide array of binding and non-binding international instruments that contribute to the protection of public health [51]. This makes generalizations difficult. The central role of health in economic development was boosted by the World Bank's *World Development Report 1993 *[52], and given further impetus by the signing of the Millennium Development Declaration in 2000 [42,53]. A variety of instruments – from conventions on hazardous chemicals, to International Labour Organisation (ILO) conventions on occupational health and safety, to various codes and provisions on infant nutrition – answer to concerns about non-communicable disease [51]. Despite this, the attention that "lifestyle-related", non-communicable diseases have received, both in health development and in international law, is grossly disproportionate to the share of global death and disability that they represent.

The most significant health development program within the United Nations system is the Millennium Development Goals (MDGs). Health is a focus of three of the eight goals ("reduce child mortality"; "improve maternal health"; "combat HIV/AIDS, malaria and other diseases"), and improvements in health would also advance those goals relating to the eradication of extreme poverty and hunger, education, and gender equality [54]. WHO points out that "the health priorities reflected in the MDGs – HIV, malaria, TB and other communicable diseases along with maternal deaths – together account for 32% of global mortality" [54]. While health is clearly central, therefore, to the UN development agenda, non-communicable diseases are conspicuously absent from the MDGs.

WHO's involvement in non-communicable diseases is relatively recent and spans both legal and non-legal processes. WHO has a broad treaty-making power with respect to matters within its competence, and regulations made by the World Health Assembly bind Members on an opt-out basis [55]. For most of its history, however, WHO has focused on the provision of expert policy advice to governments and neglected to use its law-making powers [56,57]. As noted above, this changed in 1998 when – under the leadership of its Director-General – WHO prioritized tobacco control and began negotiations towards a framework convention. The FCTC was endorsed by the World Health Assembly on 21 May 2003 and entered into force on 27 February 2005 [20,58]. It currently has 146 signatories.

In January 2000, WHO established a Commission on Macroeconomics and Health to inquire into the role of health in global economic development. The Commission's report focused substantially on investment in maternal and perinatal health, communicable diseases and tobacco-related diseases [59]. The Commission's key recommendations related to financing the scaling up of access to essential health services, and to improved access to medicines for the communicable diseases that disproportionately burden poorer countries [59]. The Commission's focus on the Millennium health goals, plus tobacco, gives implicit support to the view that communicable diseases matter more to the poor than non-communicable ones, and that the significance of non-communicable conditions only increases as poverty recedes [[60], cf. [61]]. This might be true of sub-Saharan Africa, but it is not true of countries such as Russia, where life expectancy is falling and the population is shrinking due to the catastrophic impact of cardiovascular disease, cancer, and injuries [62]. Reducing the impact of cardiovascular disease, injuries and violence in Russia to EU levels would improve life expectancy by over 10 years, as compared to a gain of 0.88 years for reducing infant, child and maternal mortality to EU levels [63].

Apart from the FCTC, WHO's main contribution in the area of chronic, lifestyle-related diseases is its *Global Strategy on Diet, Physical Activity and Health *(GSDPAH) (2004) [21,64]. The GSDPAH adopts a facilitative, advocacy-based approach. It builds on a brief, earlier strategy that called attention to the role of tobacco, unhealthy diet and physical inactivity in the most prominent non-communicable diseases: cardiovascular disease, cancer, chronic obstructive pulmonary disease and diabetes [65,66]. The GSDPAH synthesises evidence for action and sets out the key elements of a policy framework. It identifies the assistance WHO is able to provide, together with the roles of member states in implementing national policies, and the respective contributions of other global policy actors, civil society, and the private sector. Governments have a broad role that encompasses coordination of policy across various sectors and ministries; the provision of accurate information and regulation of marketing, labeling, and health claims; fiscal and agricultural policies; and promotion of physical activity. The *Global Strategy *envisages close cooperation with other UN agencies, the WTO, World Bank, other development banks and the Codex Alimentarius Commission [21].

In May 2006, progress towards implementing the *Global Strategy *was described as "limited by resource constraints, both human and financial" [22]. By January 2007, twenty-five countries had implemented (certain) policy options recommended by the Strategy, and 17 were planning to do so [23]. This disappointing assessment reflects "continuing low investment in prevention and control of chronic, noncommunicable diseases at local and global level" [22]. In Europe, progress on non-communicable diseases has been assisted by the recent adoption, by EU Ministers, of a *European Charter on Counteracting Obesity *[67]. The *Charter *was drafted by the WHO Regional Office for Europe and provides guiding principles and a policy framework for EU countries. It calls for "visible progress" within the next 4–5 years, and a reverse in rates of obesity among children and adolescents by 2015 [67].

The World Bank remains one of the largest multilateral investors in the health sector, committing US$13.9 billion to Health, Nutrition and Population (HNP) projects between 1997–2006, roughly split between Bank lending and concessional IDA assistance [68]. The Bank's involvement in health has developed over time and remains controversial [69,70], partly due to its pursuit of "Washington consensus" policies during the 1990s [71-73]. Since 1999, the World Bank and International Monetary Fund (IMF) have both sought to elicit greater political commitment, stakeholder participation and country ownership of development strategies by requiring all countries seeking debt relief or concessional assistance to prepare Poverty Reduction Strategy Papers (PRSPs) that identify development goals and the policies, programs and resources needed to achieve them [74,75].

The Bank enjoys several advantages as a global health policy actor. These include its global experience, strong country presence, capacity to engage with all government sectors including Finance Ministries, and its capacity for large-scale program implementation including financing and financial management [68]. This gives the Bank a unique capacity to engage with the determinants of non-communicable disease from a system-wide perspective within client countries. Under the 1997 HNP strategy, the Bank has focused on health systems development, health care financing, and improving the health, nutrition and population outcomes of the world's poor [76]. In practice, priority in lending and policy dialogue has gone to achieving the MDGs [77]. Work towards the 2007 HNP strategy does not suggest any major departure from this approach, although the Bank acknowledges that non-communicable diseases have become a "critical challenge" due to "an epidemic of obesity and tobacco consumption" in most middle income and many lower income countries [68]. Given the proliferation of entities financing single diseases and vaccines, the Bank has signaled a greater focus on strengthening health systems as a whole as a way of achieving its other health objectives [68]. This strategic focus is important: to be capable of responding effectively to chronic diseases, health systems must be capable of providing continuity of care in the community, with services encompassing primary and secondary prevention, monitoring of risk factors and treatment of chronic illness [41,78].

In summary, communicable diseases and the MDGs remain the priority of the leading global agencies investing in health. The FCTC has improved the visibility of tobacco as the world's leading cause of preventable disease, but obesity, diet and physical activity are yet to achieve priority status, despite growing policy interest in obesity in developed countries. Over the past decade, private foundations and public/private partnerships have injected significant new funding into the health sector. This has mostly been directed to priority communicable diseases (HIV/AIDS, malaria, TB) or to other vertical programs (vaccinations, essential medicines), rather than to chronic diseases initiatives [68,79]. Despite the much-touted role of civil society and the private sector in global health governance, their capacity to refocus and coordinate global health strategies should not be over-stated. Global health agencies need to be centrally involved with chronic diseases, providing "institutional focal points for global debate", political mobilization, and law-making [40].

## What can we learn from the Millennium Development Goals and the Framework Convention on Tobacco Control?

The Millennium Development Goals (MDGs) and the Framework Convention on Tobacco Control (FCTC) reflect two different processes for advancing global health policies. The FCTC is an evidence-based treaty that identifies core areas of consensus over regulatory measures that signatory countries are legally required to implement within their domestic systems. The MDGs are a global partnership embracing ambitious goals to be achieved collectively within a 15 year timeframe (2000–2015). In addition to legal obligations, and partnerships involving global and national stakeholders, economic pressure and funding conditionality – historically associated with World Bank lending – provide a third process for encouraging policy change at country level.

### A global political partnership: the Millennium Development Goals

The remarkable achievement of the Millennium Declaration was to "re-package" key global challenges into a single cluster of political commitments, while delivering the crucial coordinating role to the Secretary-General to translate the commitments into a coherent work program, and to report periodically to the General Assembly [42,80]. From the beginning, work on the MDGs has sought to strengthen linkages with a wide range of stakeholders, including other international agencies within and beyond the UN system [81]. By locating the executive functions directly within the UN Secretariat, the MDGs found a high profile "political champion" and *became *the preeminent development priority within the United Nations system. This political impetus has been significantly strengthened by the commitment, technical expertise and/or financial backing of partner institutions, including WHO and the World Bank.

This is not to deny that there have been setbacks in progress towards the MDGs. While financial and human resource constraints are a factor, effective interventions need to be better targeted to the poor, policies need to focus on prevention and demand for health care at the household level, and health systems need strengthening overall [82]. No process can guarantee success. The scale of the challenge, however, demonstrates the need for a partnership approach. Provided there is sufficiently close alignment between "partnership aims" and the core functions of each agency, partnerships multiply the resources available for furthering partnership goals, and the opportunities for policy influence at country level. While the "MDGs do not reflect the entirety of WHO's work", they are "central to its agenda...and represent important milestones against which the Organization's overall contribution to health development can be measured" [54]. Similarly, although World Bank activities extend well beyond health, there is a natural fit between " [t]he first seven Millennium Development goals" and "the activities of the health, nutrition and population sector in the World Bank, either as health and nutrition status indicators or as determinants of health outcomes" [83].

### Binding global norms: The Framework Convention on Tobacco Control

The success of multinational tobacco corporations in penetrating developing country markets due to mergers and trade liberalization, and the resulting globalization of the tobacco epidemic, have galvanized support for the development of global norms for tobacco control. International legal agreements have been recognised as "global public goods" that strengthen national capacity to achieve public health goals by adopting a unified response to transnational threats [19,84]. International law has a direct impact on domestic laws and policies because of the obligation that countries accept, upon ratification of a treaty, to implement its provisions. The intention behind the FCTC is that parties to the treaty will regulate their domestic tobacco economies and impose legal constraints on the activities of tobacco manufacturers and retailers.

The FCTC reflects a framework-protocol approach. It sets out "baseline international norms", together with institutional arrangements for global governance of tobacco: provisions relating to a permanent Secretariat and periodic conference of the parties, the exchange of information, reporting and technical cooperation, and the financing of Convention objectives. The framework-protocol approach was a strategic choice made in the light of concerns about loss of sovereignty and trade, pressure from the tobacco industry, and the evolving evidence base about harm from exposure to tobacco [19,20,48,85]. The later development of protocols provides scope for the deepening of international standards as political will hardens in response to the success of tobacco control policies, reduced smoking prevalence, and waning industry influence [86]. A feature of the FCTC was the participation of NGOs during the negotiations and their ongoing input into the conference of parties [87,88]. The FCTC also provides a mandate for the development of global standards for regulating the constituents of tobacco products. This is a novel feature of the Convention that adds harm reduction measures to the more traditional supply and demand controls [20].

As a convention negotiated in the shadow of tobacco trade liberalization and industry influence, the evidence base for tobacco control was critical to the political legitimacy of the FCTC process. This will remain the case in future, whenever global health standards challenge economic and business interests. Public health advocates are divided over the potential for World Trade Organisation (WTO) agreements to undermine the capacity of national governments to protect public health [89-92]. The FCTC provides a mandate for domestic tobacco control legislation through a global treaty negotiated outside the WTO process. It makes better sense for WHO to act assertively as a global policy actor in its own right and to coordinate the development of new legal standards in a forum "where public health issues predominate", than to seek to carve out new exemptions for health from the WTO [89]. The WTO is neither a scientific nor a health agency and it does not develop standards [93]. The shaping of global health standards is therefore best achieved through WTO input into WHO processes, rather than the other way around. The recent WTO Panel Report in the *EC-Biotech Products *case suggests that WTO disputes are likely to be resolved on narrow terms, with little regard to other international obligations not assumed by all disputing parties (if not all WTO members) [94]. As discussed below, the development of global health standards, in a manner consistent with WTO obligations, provides the best protection for countries who wish to avoid the threat of trade disputes arising from their domestic public health policies.

The FCTC has brought considerable prestige to WHO. At the same time, the FCTC process lacks the "shared ownership" of global policy goals that characterizes the UN/WHO/World Bank response to the MDGs. The mutually reinforcing influences made possible through partnerships could significantly assist an agency such as WHO, which has limited resources of its own, and whose political influence is centred on Health Ministries. In this respect, New York Mayor Michael Bloomberg's historic donation of $125 million towards tobacco control efforts in low and middle income countries, provides interesting opportunities for new partnerships to evolve in tobacco control [95].

### WTO rules and international health standards

As the FCTC illustrates, one important way of minimizing the scope for conflict between global health goals and trade obligations is to *develop international health standards*. Under the *General Agreement on Tariffs and Trade *(GATT), WTO members are required not to adopt measures that discriminate between the imports of different countries, or between domestic goods and imports. These are the "Most Favoured Nation" rule, and the "National Treatment" rule in GATT Articles I, and III, respectively [91,96]. GATT contains an exception to these rules for measures "necessary to protect human, animal or plant life or health" (Art XX(b)) [96].

The scope of the exception is clarified by the WTO *Agreement on the Application of Sanitary and Phytosanitary Measures *(SPS Agreement). The SPS Agreement applies to "SPS measures", including domestic laws or policies applied to protect human life and health from additives, contaminants, toxins or disease-causing organisms in foods and beverages. Where they exist, the SPS Agreement requires members to base their SPS measures on international standards, guidelines or recommendations [97]. Member states whose SPS measures conform to international standards are deemed to be acting consistently with both the SPS Agreement itself, and GATT Article XX(b). Member states whose SPS measures exceed the requirements of international standards, however, must demonstrate that those standards are supported by and do not exceed the scientific evidence, and are based on an assessment of the risks to human life or health [97].

The WTO *Agreement on Technical Barriers to Trade *(the TBT Agreement) applies to technical regulatory measures that are not covered by the SPS Agreement [98]. Under the TBT Agreement, member states must comply with the "Most Favoured Nation" and "National Treatment" rules. They must also ensure that technical regulations do not create "unnecessary obstacles to international trade", by being "more trade-restrictive than necessary to fulfil a legitimate objective". Where they exist, member states must base their domestic standards on international standards, and when applied for a legitimate objective, such standards will be "rebuttably presumed not to create an unnecessary obstacle to international trade" [98]. The legitimate objectives include preventing deceptive practices, and the protection of human health or safety [98]. Although the SPS and TBT Agreements can not apply to the same measure, the WTO Panel report in the *EC-Biotech Products *case suggests that specific requirements embodied in a domestic law (in this case an EU law) can themselves be considered to embody *both *an SPS and a TBT measure, depending on the purpose for which they are applied [94]. This suggests that domestic laws enacted in accordance with TBT provisions might withstand scrutiny even if they failed as SPS measures.

The "human health" exceptions in WTO rules provide a space for the development of global health norms as a way of overcoming "regulatory chill": the reluctance of governments to introduce domestic public health laws for fear of inviting trade disputes. Indeed, the development of evidence-based international standards perform a valuable function. As noted above, domestic laws based on international standards are deemed to satisfy SPS obligations and will presumptively satisfy TBT obligations. Furthermore, when member states introduce SPS measures in the absence of international standards, or where those measures go beyond international standards, they bear the onus of demonstrating that each measure is reasonably supported by a risk assessment as defined in the Agreement [97]. Countries seeking to protect the health of their populations are therefore well served by global agencies that are actively engaged in standards development.

## Leveraging the *Global Strategy on Diet, Physical Activity and Health*

The Millennium Development Goals and the *Framework Convention on Tobacco Control *serve as helpful models when considering ways of strengthening the global response to non-communicable diseases.

### Global targets for reductions in chronic diseases?

At first glance, WHO's *Global Strategy on Diet, Physical Activity and Health *(GSDPAH) shares similarities with the MDG process. WHO is the obvious "political champion" for the strategy, which proposes: "an ad hoc committee of partners within the United Nations system", as well as links with NGOs and the private sector [21].

The MDG model suggests that the challenge for WHO, if adopting a partnership approach to the GSDPAH, is to successfully link implementation to the core functions – *and resources *– of partner agencies. One way to achieve this is to integrate non-communicable diseases into an existing global health partnership. Commentators have suggested that the MDGs should expand to include non-communicable disease targets. There have been calls to broaden the child mortality focus of the MDGs to include adult mortality and morbidity, either as a separate goal in its own right, or as a target falling under Goal 6, which aims to "combat HIV/AIDS, malaria *and other diseases*" [29,63,99]. The contribution that tobacco control policies can make to the MDGs has been emphasized [54,100], and commentators have also stressed the link between chronic diseases and poverty reduction [101].

The Millennium Development Goals have achieved broad acceptance as a framework for measuring and achieving social development. This may be reason enough for caution in tinkering with them, or seeking to re-cast them mid-stream. An additional set of UN-sponsored health targets could undermine the status the MDGs have managed to achieve as global priorities and aggravate existing difficulties in progress towards them [54,82,102,103]. The UN Secretariat is fully invested in the MDGs and unlikely to take on new responsibilities around chronic diseases.

This has not stopped public health advocates, and WHO itself, from advocating a global goal of reducing death rates from chronic diseases by 2% annually, resulting in 36 million fewer deaths by 2015 [27,29]. The difficulty is that goals are ends, rather than means [102]. To achieve global goals requires the simultaneous action of many countries: each making budgetary commitments, implementing policy changes, and monitoring outcomes in order to achieve real results on the ground.

The importance of linking global goals to legal, economic, or multilateral political processes can be illustrated by contrasting WHO's Commission on Macroeconomics and Health with World Bank concessional (IDA) credits. In its 2001 report, the Commission recommended the establishment of national commissions to lead the process of scaling up access to essential health services [59]. This process has also been identified as providing the opportunity to include strategies for cardiovascular disease [100], and to adapt MDG targets to the health priorities of individual countries [104]. By 2006, only 20 countries had established national commissions or used existing bodies to strengthen health policy reform [104].

For World Bank borrowers, the Poverty Reduction Strategy Paper (PRSP) process requires countries seeking concessional financing to identify their national development priorities in a PRSP. The Bank and the IMF exercise considerable real-world influence over these country-level priorities. In a recent review, both agencies echoed a call by the UN Development Program to use the PRSP process as a vehicle for scaling up country-level efforts to achieve the MDGs [53,74,75]. The PRSP process could also provide an operational framework for setting out the strategies required to meet national goals on non-communicable diseases. The advantage of the PRPS process is the economic incentive it creates to take concrete actions in support of national priorities.

### Appropriate global partners?

The advantages of a partnership approach to global health challenges include greater access to expert input and advocacy from policy partners, greater publicity for policy goals, and greater opportunity for engaging at country level beyond traditional WHO-Health Ministry relationships. The GSDPAH identifies a wide range of potential partners including ECOSOC, the ILO, UNESCO, and the WTO. There are some clear synergies; for example, with the Food and Agricultural Organisation (FAO) around promoting the supply and consumption of fruit and vegetables [105], and with UNICEF, around children's diets, nutrition education, and food marketing. Given its strong human rights focus, UNICEF could be an important ally in advocating a restrained approach to industry marketing, reprising the role if played during development of the *International Code of Marketing of Breast-Milk Substitutes *[106].

The Codex Alimentarius Commission, as the body responsible for developing global food standards, has been identified by WHO as an important partner in the GSDPAH [21]. This partnership is significant since countries implementing Codex standards are presumed to be acting consistently with GATT and the SPS Agreement [96,97]. FAO/WHO have highlighted the role that the *Codex Committee on Food Labeling *might play in developing guidelines on the use of consumer-friendly nutrition labeling and health claims [107]. They have also pointed to the role that the *Codex Committee on Nutrition and Foods for Special Dietary Uses *could play in developing food composition standards [108]. Codex is awaiting a joint WHO/FAO paper outlining concrete proposals that it might take [109].

It is highly uncertain whether the involvement of Codex will contribute to progressive global standards, given its reputation for being dominated by industry [110,111]. Historically, Codex – like national food regulators – has focused on food safety, rather than nutritional quality. On one view, there is limited scope for WHO to address issues falling within the Commission's authority, since conflicting WHO/Codex standards could create confusion, and might undermine WHO's position, given the status of Codex standards under WTO rules [112]. However, this criticism could also be made of WHO's marketing code for Breast-Milk Substitutes [106]. Rather than risk abandoning important areas of diet and nutrition to Codex, WHO could make a virtue out of Codex' technical and historically more limited role, seeking Codex input while nevertheless retaining ownership of the key areas of labeling, health claims, and food composition standards for trans and saturated fats, salt and free sugars. As with the FCTC, there is strategic benefit in locating standards development within a forum where health issues predominate.

As noted above, the World Bank could be an important partner for implementing the GSDPAH, especially given its greater financial resources. There are several areas where the GSDPAH overlaps with World Bank investments in health, nutrition and population (HNP). The first is in tobacco, where the Bank's work on the economic benefits of tobacco control policies, including tobacco taxes, complements WHO's desire to encourage implementation of the FCTC [113]. Secondly, the Bank has staked out nutrition as a priority. The Bank acknowledges that obesity is part of the nutrition policy agenda [114], and diet-related interventions are a cost-effective way of preventing cardiovascular disease [9,115]. As with tobacco, it makes sense to pool WHO's technical and policy experience with the Bank's resources and country-level knowledge wherever possible. Thirdly, the new HNP strategy for the Bank makes public health surveillance and health system performance monitoring a priority [68]. Besides permitting better evaluation of the Bank's own programs, enhanced surveillance cannot but call attention to the growing burden of chronic disease. Finally, as noted above, the Bank could require concessional borrowers to address non-communicable diseases within their Poverty Reduction Strategy Papers.

While partnerships between international agencies primarily enhance links with government, NGOs and civil society networks have a broader role. Besides pressuring governments to implement healthy policies, that role can extend to pressuring the private sector for access to healthier foods, sharing information, and influencing consumers directly in ways that reduce lifestyle risk factors and shape market demand [116]. The GSDPAH expresses high hopes for partnerships with civil society, NGOs and the private sector, but their assistance in advancing the strategy appears to be modest so far [22].

### Are partnerships with the processed food industry a good idea?

In contrast to the tobacco industry, WHO has welcomed the food industry as a partner, citing its capacity to develop healthier products and to encourage healthier choices [21,117]. This is a high-risk strategy, given well-publicised attempts by the United States, acting on behalf of its sugar industry, to weaken the GSDPAH during its development [47,118]. In 2006, a study of global food manufacturers, retailers and food service companies concluded that only a minority had altered their business practices in response to the GSDPAH. Of the 25 corporations studied, ten had taken action on salt, five on sugar, four on fat and eight on trans fats, but only two on portion sizes [119]. In Europe, the *EU Platform for Action on Diet, Physical Activity and Health *has, since March 2005, provided a forum for the food industry, as well as NGOs, medical and consumer groups, to make public commitments on measures to reduce obesity and to improve diet and physical activity [120-122]. For example, nine soft drink makers have undertaken not to advertise soft drinks to children *aged 11 or less*, reaping high praise from the European Health and Consumer Protection Commissioner [123]. In the United States, the Alliance for a Healthier Generation, a joint initiative of the American Heart Association and the William J. Clinton Foundation, has negotiated voluntary agreements with three large beverage markers, and five large snack food makers, to comply with new school beverage and competitive food guidelines in their marketing to 123,000 schools nationwide [124-127].

If partnerships between global health agencies, governments, and the private sector yield tangible outcomes, so much the better. Pursuing voluntary commitments is cheaper and faster than the difficult process of standards-setting. However, short-term gains should not be confused with the trans-generational challenge of improving global diets. At the population level, a reduction in the burden of chronic disease requires reduced consumption of high-sugar, high-fat, high-salt foods, and increased consumption of fresh fruit and vegetables – in most countries in the world.

The dilemma for the processed food industry is that "'good' foods are bad commodities with low profit margins while 'bad' foods are good commodities with high margins" [128]. It is difficult for consumer demand to drive industry improvements when that demand is itself skillfully manipulated by vast advertising expenditures exceeding WHO's annual budget many times over [119]. It is perfectly rational for global food companies to cooperate to the degree necessary to enhance their reputations and to avoid threats of regulation while nevertheless protecting established markets for energy-dense foods of poor nutritional value.

### Should there be global legal standards on diet and nutrition?

Leading public health advocates have shown little enthusiasm for using treaties to progress a global strategy on diet, nutrition and physical inactivity [5,129,130], although this view has been challenged [88,131,132]. WHO's preference for a voluntaristic approach may rest on several assumptions: that partnering with industry is the most effective way of aligning commercial incentives with consumer health interests, that regulation will destroy communication channels with business, and that regulatory options are not politically feasible and could backfire. WHO's reluctance may also reflect the fact that the determinants of nutrition and obesity-related disease are complex and cannot easily be reduced to legal principles, and perhaps that the evidence base for intervention is less robust than in tobacco control.

Like tobacco, nutrition and obesity-related diseases develop over a considerable period of time. Commentators point out, however, that unlike tobacco, food is not hazardous per se, except when perished or adulterated [133]. This distinction directs attention away from the constituents of individual foods and beverages, towards lifestyles and dietary choices. The food industry argues that the solution lies with the individual (more physical activity, wiser food choices), assisted by wider product choice where consumer demand makes this commercially viable. What cannot be denied is that if high-fat, high-salt, high-sugar foods are "hazardous when consumed [too] frequently" [133], then reducing the hazard means either altering the products that are (over)consumed, thereby potentially interfering with their market appeal, or intervening with supply and demand in ways that reduce consumption levels. Each one of these strategies: changing product composition, influencing demand for, or regulating the availability of, food products, puts markets at risk.

Voluntary commitments by industry are welcome and should be accepted whenever offered. But public health stakeholders should be aware that business corporations are not constituted to act sacrificially in pursuit of worthy aims. "Forbearance" from pursuing certain kinds of sales opportunities may prevent damage to – or even enhance – a company's reputation. To that extent, investments in corporate social responsibility can bring economic benefits. As Redmond points out, business corporations may engage in social and human rights entrepreneurialism, competing for consumers or investors "by means of signaled respect for human rights standards in company operations" [134]. Corporations may also embrace voluntary codes and principles to forestall the risk of more intrusive or costly forms of regulation in future [135]. Company executives may be well motivated and keen to make the world a better place. But corporations have little incentive to re-shape public tastes and existing product lines, as distinct from offering marginally "better for you" variants, when doing so risks sacrificing existing markets and provides opportunities for competitors. Regulation has the advantage of creating a level playing field; while food companies may not like regulation, they understand it and will usually absorb it into their operations as efficiently as they can.

Clearly, not all aspects of diet, nutrition and physical activity are appropriate subjects for legal standards, even aspirational or broadly-stated ones. For example, physical activity and food choices at the individual level are a matter for personal choice, except in schools. Food security, and the provision of fresh fruit and vegetables at affordable prices, are more likely to be a matter for national policy and coordination across the agricultural, finance, trade, employment, transport and health sectors, rather than legal prescriptions. Strategies to encourage physical activity and healthy eating may also, appropriately, vary from country to country. There are four areas, however, where the development of global standards could leverage the *Global Strategy *beyond a purely voluntary menu of policy options for governments.

The first area relates to the informational basis for making informed and healthy food choices: standards for the labeling of product constituents, fair warning of health risks, and health claims. Accurate information in rapidly-digestible formats could enhance competition for healthy products. In so far as markets have failed to provide consumers with sufficient information, or have perverse incentives to obscure such knowledge, regulation may be justified as a "public good" [136].

Domestic laws implementing global standards in this area would need to be consistent with that country's WTO obligations. As noted above, the WTO Panel report in the *EC-Biotech Products *case suggests that a particular legal requirement might be considered to embody both an SPS and a TBT measure, although justification of the measure under either Agreement will be sufficient [94]. To the extent that national laws embodying or requiring compliance with international standards on labeling were enacted in order to inform consumers, or to avoid confusion or misleading impressions, they would be "rebuttably presumed not to create an unnecessary obstacle to international trade", under the TBT Agreement [98].

In some cases, labeling requirements and health warnings might also be characterized as intending to warn consumers of the *health risks *of over-consumption of product ingredients, such as salt, sugar or fat. The SPS Agreement applies to measures that are intended to mitigate risks to human life or health arising from "additives, contaminants, toxins or disease-causing organisms". In the *EC-Biotech Products *case, the Panel distinguished between foods that posed a danger to the life or health of the customer, and foods that were nutritionally disadvantageous due to the quality or quantity of their nutrients, but without necessarily presenting a danger to the health of the consumer. The Panel made it clear that the SPS Agreement only applies to laws seeking to address a "danger for the consumer" [94].

This approach supports the argument that laws seeking to mitigate the risks of harmful diets by warning consumers about the (poor) nutritional quality of a food, would not be SPS measures, although they might well fall for consideration under the TBT Agreement. It follows that the question of whether salt, sugar or fat can be described as an "additive" creating a risk to human health, within the terms of the SPS Agreement, will not arise. In any event, the Panel interpreted "food additive" to mean a substance "not normally used as a typical ingredient" in food, which would seem to exclude sugar, salt and fat [94]. It seems likely that future WTO Panels would follow this interpretation, since it avoids framing diet-related harms in terms of the intrinsic properties of salt, sugar and fat in food, as distinct from their nutritional impact over time. The end result is that health warnings about the nutritional quality of food are likely to be treated as TBT measures.

The second area for the development of international standards relates to food composition. Estimates from the Global Burden of Disease Study indicate that high blood pressure, high cholesterol, and overweight and obesity, respectively, were responsible for 46%, 24%, and 11% of global mortality from cardiovascular disease in 2001, and almost equivalent shares of CVD morbidity [137]. There is an established relationship at the population level between salt intake and blood pressure, and between dietary fats and cholesterol, blood pressure and weight [9]. Applying the distinction made by the Panel in the *EC-Biotech Products *case, evidence-based regulations or recommendations on food composition, based on dietary risks to health at the population level would likely be treated as TBT measures. National legislation implementing such measures would bind multinational food corporations and other food producers, and could improve dietary balance by reducing the intake of trans and saturated fats, salt, and sugar, at the population level.

A third possible area for legal intervention relates to children's health, nutrition and education and, more controversially, food marketing to children. Despite the reluctance of many governments to interfere with market processes, there is wide acceptance that the vulnerability of children supports protective regulation. WHO's *International Code of Marketing of Breast-milk Substitutes *already bans promotion of breast-milk substitutes [106]. A recent WHO-sponsored forum and technical meeting concluded that self-regulation of food and beverage marketing directed at children is unlikely to be effective, and recommended that WHO develop an international code addressing promotional activities by transnational companies irrespective of country [138]. In so far as "the origins of obesity and NCDs such as cardio vascular heart disease and diabetes...lie in early childhood", regulations aimed at enhancing children's nutrition would be highly cost-effective [114,139].

A fourth area for global standards relates to surveillance of chronic disease risk factors and the obligation to report on progress in implementing policies on non-communicable diseases. Reporting provisions can help to maintain commitment towards tackling longer-term problems and enhance the implementation of both legal and non-legal aspects of a global strategy. Periodic reporting also provides a focal point for the participation of civil society [85].

One criticism of the GSDPAH and of WHO's framework for implementation [140] is that it anticipates purely voluntary measures at a time when *developed *countries are debating and experimenting with legal strategies to combat obesity and chronic diseases in precisely the areas identified above. For example, Denmark has outlawed the use of trans fats in food [141]. New York City has banned artificial trans fats in restaurant cooking [142,143], and requires the provision of calorie information on restaurant menus [144]. The Food Safety Authority of Ireland is negotiating salt commitments with food businesses [145], while in the United States, public health bodies are lobbying for the Food and Drug Administration (FDA) to regulate salt as a food additive [146-148]. Britain's telecommunications regulator, Ofcom, has banned television advertising of foods assessed as high in fat, salt or sugar during children's programs [149,150]. Similarly, the *European Charter on Counteracting Obesity *specifies that EU governments should adopt specific regulatory measures to "substantially reduce the extent and impact of commercial promotion of energy-dense food and beverages" to children, moving towards an international code of practice in this area [67]. The United States is a social laboratory for a variety of legal responses to obesity [151,152]. Congress has imposed conditions on federal grants funding school breakfast and lunch programs that require compliance with nutrition guidelines and nutrition education within the curriculum [153-155].

The use of law as a process for developing global standards for diet and nutrition needs to be distinguished from the use of law as a process for implementing those standards at the domestic level (through legislation). Ultimately, whether developed countries pass domestic laws in the areas identified above, or secure the health of their populations in other ways, is not the point. The point is that global standards provide a baseline for responsible transnational corporate behaviour. Appropriately implemented at country level, binding international standards on diet and nutrition will make the health of future generations less dependent upon corporate charity and voluntary commitments. It will also reduce the "regulatory gap" that might otherwise emerge between developed and developing countries, due to the vulnerability of the latter to pressure from large transnational corporations.

### If not a treaty on diet and nutrition, then what?

Regulatory approaches to diet and nutrition need not imply the replication of the FCTC approach to the food industry [130]. Any attempt to mirror the FCTF approach by consolidating the regulation of food composition, labeling, health claims, food advertising and children's health and nutrition into a single instrument would likely fail due to political divisions and industry opposition. While progress on all these fronts is desirable, each element also has value in itself. By keeping issues separate; for example, by developing separate standards for salt, fats, and sugar, labeling, and marketing, WHO could more effectively maintain the focus on evidence in each case, and limit engagement to those whose interests were directly affected by the standards in each case. One hopes, for example, that an instrument on salt would not be thrown off course by lobbying for and on behalf of the sugar industry. This multi-track approach need not, however, limit the influence of NGO and consumer groups. Issues where the evidence is strongest should be tacked first. All issues should be framed as components of a broader global strategy on non-communicable diseases comprising "hard law", "soft law", and purely recommendatory elements.

WHO has an unambiguous constitutional mandate to "develop, establish and promote international standards with respect to food, biological, pharmaceutical and similar products" [55]. While WHO's treaty-making power is expressed in general terms to extend to any matter within its competence, its power to make regulations extends to specific, enumerated areas [55,132]. WHO's regulations power extends to making advertising and labeling standards, and standards with respect to the safety, of "biological, pharmaceutical and similar products moving in international trade" [55]. If "similar products" includes food, then WHO's power to make regulations in these areas seems assured; on balance, however, a narrower interpretation seems likely. A more promising basis for developing standards on diet and nutrition depends on whether such standards come within the terms of: "sanitary and quarantine requirements and *other procedures designed to prevent the international spread of disease*" (Article 21(a)). If "disease" is given a narrow meaning, to exclude chronic, diet-related diseases, then WHO would be required to either use its treaty power (Article 19), or to make non-binding "recommendations" (Article 23). Ultimately, the scope of Article 19 will depend on whether a political consensus can be reached among World Health Assembly members.

At the present time, it may seem novel to talk about national and international laws for fighting heart disease, diabetes and other chronic diseases. However, non-communicable diseases are highly unlikely to remain a "law-free zone" over the medium term. Even assuming that no consensus can be reached on the use of WHO's treaty or regulations power, the development of recommendations preserves the obligation of states to report to WHO periodically (Article 62), and WHO could publicly state its aspiration to consolidate recommendations in this area into a legally binding instrument at a later date.

In summary, the opportunities for using international law as a process for implementing the GSDPAH have been underestimated. The transnational factors influencing dietary trends, and the degree of control that transnational corporations exercise over global diets, support a global standards approach. At the same time, diet and nutrition are more complex and nuanced than tobacco. Law is an important global process, but not the only one. The development of legal standards in key areas could strengthen a wider strategy on non-communicable diseases comprising legal and policy elements, with careful use of partnerships and economic incentives.

## Conclusion

Non-communicable diseases are a serious threat to health in both developed and developing countries and deserve to be treated as a global health priority. Enough is known at the policy level to make a significant impact on the burden of disease. There is a persistent gap, however, between policy knowledge at the global level, and policy implementation at country level. This paper has highlighted the role that global processes can play in generating the political commitment to narrow that gap.

The key lifestyle risks for non-communicable diseases have been clearly identified: tobacco use, physical inactivity, and the over-consumption of nutrient-poor foods that contain too much fat, salt, and sugar [21,29]. These factors cluster together in their impact on key non-communicable diseases [137]. It makes sense, therefore, to link the FCTC and GSDPAH together as core components of a broader global strategy on non-communicable diseases.

The implementation of a global strategy on non-communicable diseases requires intervention in sectors and policy settings that extend well "beyond the traditional mandates and authority of health ministries and authorities" [41]. WHO is the obvious agency to coordinate and act as political champion for a global response. Jointly-conceived strategies would also be valuable given, for example, the common interest of WHO and the World Bank in nutrition and obesity in poor countries. At the same time, care should be taken with choice of partners so as not to cede control of issues to agencies whose institutional focus could weaken global health norms. The potential for WHO to leverage its strategy by using its law-making powers deserves fresh consideration. International legal standards are not self-executing, and developing countries require the capacity to implement and enforce them. However, legal standards also have a normative role, compliance can be monitored by NGOs as well as governments, and a global approach could reduce the health inequalities that might otherwise result from uneven patterns of "negotiated commitments" with the processed food industry.

## References

[B1] United Nations Development Program (UNDP) (2005). Human Development Report.

[B2] Beaglehole R, Bonita R (2004). Public Health at the Crossroads: Achievements and Prospects.

[B3] WHO (2003). World Health Report 2003 Geneva, WHO.

[B4] Murray C, Lopez A (1996). Global Patterns of Cause of Death and Burden of Disease in 1990, With Projections to 2020. Annex 1 in Investing in Health Research and Development, Report of the Ad Hoc Committee on Health Research Relating to Future Intervention Options.

[B5] Yach D, Beaglehole R (2004). Globalization of Risks for Chronic Diseases Demands Global Solutions. Perspectives on Global Development and Technology.

[B6] Yach D, Leeder SR, Bell J, Kistnasamy B (2005). Global Chronic Diseases. Science.

[B7] McMichael A, McKee M, Shkolnikov V, Valkonen T (2004). Mortality Trends and Setbacks: Global Convergence or Divergence?. Lancet.

[B8] Popkin B, Maxwell S, Slater R (2004). The Nutrition Transition in the Developing World. Food Policy Old and New.

[B9] WHO (2003). Diet, Nutrition and the Prevention of Chronic Diseases WHO Technical Report Series 916 Geneva, WHO.

[B10] WHO (2000). Obesity: Preventing and Managing the Global Epidemic WHO Technical Report Series 894 Geneva, WHO.

[B11] Hawkes C (2006). Uneven Dietary Development: linking the policies and processes of globalization with the nutrition transition, obesity and diet-related chronic diseases. Globalization and Health.

[B12] Mendez M, Popkin B (2004). Globalization, Urbanization and Nutritional Change in the Developing World. e-Journal of Agricultural and Development Economics.

[B13] Popkin B (2005). Using Research on the Obesity Pandemic as a Guide to a Unified Vision of Nutrition. Public Health Nutrition.

[B14] Lang T, Maxwell S, Slater R (2004). Food Industrialisation and Food Power: Implications for Food Governance. Food Policy Old and New.

[B15] Yach D, Stuckler D, Brownell K (2006). Epidemiologic and Economic Consequences of the Global Epidemics of Obesity and Diabetes. Nature Medicine.

[B16] Drewnowski D (2000). Nutrition Transition and Global Dietary Trends. Nutrition.

[B17] Bolling C, Gehlhar M, Regmi A, Gehlhar M (2005). Global Food Manufacturing Reorients to Meet New Demands. New Directions in Global Food Markets.

[B18] Gehlhar M, Regmi A, Regmi A, Gehlhar M (2005). Factors Shaping Global Food Markets. New Directions in Global Food Markets.

[B19] Taylor A, Bettcher D (2000). WHO Framework Convention on Tobacco Control: A Global "Good" for Public Health. Bulletin of the World Health Organization.

[B20] WHO WHO Framework Convention on Tobacco Control WHA561 (entered into force 27 February 2005).

[B21] WHO Global Strategy on Diet, Physical Activity and Health WHA5717 (22 May 2004).

[B22] WHO Implementation of Resolutions (Progress Reports): Report by the Secretariat A59/23 (World Health Assembly, Provisional Agenda Item 1117; 11 May 2006).

[B23] WHO Report by the former Acting Director-General to the Executive Board at its 120th Session EB120/40 (Executive Board, 120th Session, Agenda Item 2).

[B24] Lopez A, Mathers C, Ezzati M, Jamison D, Murray C (2006). Global Burden of Disease and Risk Factors.

[B25] Lopez A, Mathers C, Ezzati M, Jamison D, Murray C (2006). Global and Regional Burden of Disease and Risk Factors, 2001: Systematic Analysis of Population Health Data. Lancet.

[B26] Leeder S, Raymond S, Greenberg H, Liu H, Esson K (2004). A Race Against Time: The Challenge of Cardiovascular Disease in Developing Economies.

[B27] Strong K, Mathers C, Leeder S, Beaglehole R (2005). Preventing Chronic Diseases: How Many Lives Can We Save?. Lancet.

[B28] Suhrcke M, Nugent R, Stuckler D, Rocco L (2006). Chronic Disease: An Economic Perspective.

[B29] WHO (2005). Preventing Chronic Diseases: A Vital Investment Geneva: WHO.

[B30] Hossain P, Kawar B, Nahas M (2007). Obesity and Diabetes in the Developing World – A Growing Challenge. New England Journal of Medicine.

[B31] Wild S, Roglic G, Green A, Sicree R, King H (2004). Global Prevalence of Diabetes: Estimates for the Year 2000 and Projections for 2030. Diabetes Care.

[B32] Ezzati M, Lopez A (2003). Estimates of Global Mortality Attributable to Smoking in 2000. Lancet.

[B33] Guindon G, Boisclair D (2003). Past, Current and Future Trends in Tobacco Use Health, Nutrition and Population (HNP) Discussion Paper (Economics of Tobacco Control Paper No 6) Washington, World Bank.

[B34] Jamison D, Breman J, Measham A, Alleyne G, Claeson M, Evans D (2006). Priorities in Health.

[B35] Peto R, Lopez A, Koop C, Pearson C, Schwarz M (2002). Future Worldwide Health Effects of Current Smoking Patterns. Critical Issues in Global Health.

[B36] Lee K, Fustukian S, Buse K, Lee K, Buse K, Fustukian S (2002). An Introduction to Global Health Policy. Health Policy in a Globalising World.

[B37] Huynen M, Martens P, Hilderink H (2005). The Health Impacts of Globalisation: A Conceptual Framework. Globalization and Health.

[B38] Yach D, Bettcher D (1998). The Globalization of Public Health I: Threats and Opportunities. American Journal of Public Health.

[B39] Buse K, Drager N, Fustukian S, Lee K, Lee K, Buse K, Fustukian S (2002). Globalisation and Health Policy: Trends and Opportunities. Health Policy in a Globalising World.

[B40] Taylor A (2004). Governing the Globalization of Public Health. Journal of Law, Medicine & Ethics.

[B41] Greenberg H, Raymond S, Leeder S (2005). Cardiovascular Disease and Global health: Threat and Opportunity. Health Affairs.

[B42] United Nations, General Assembly United Nations Millennium Declaration UN Doc A/RES/55/2 (18 September 2000).

[B43] Shearer I (1994). Starke's International Law.

[B44] Smith C (2006). Politics and Process at the United Nations: The Global Dance.

[B45] de Senarclens P (2001). International Organisations and the Challenge of Globalisation. International Social Science Journal.

[B46] Waxman H (2002). The Future of the Global Tobacco Treaty Negotiations. New England Journal of Medicine.

[B47] Cannon G (2004). Why the Bush Administration and the Global Sugar Industry are Determined to Demolish the 2004 WHO Global Strategy on Diet, Physical Activity and Health. Public Health Nutrition.

[B48] Roemer R, Taylor A, Lariviere J (2005). Origins of the WHO Framework Convention on Tobacco Control. American Journal of Public Health.

[B49] WHO International Framework Convention on Tobacco Control WHA4917 (25 May 1996).

[B50] WHO Towards a WHO Framework Convention on Tobacco Control WHA 5218 (24 May 1999).

[B51] Taylor A, Bettcher D, Fluss S, DeLand K, Yach D, Detels R, McEwen J, Beaglehold R, Tanaka H (2004). International Health Instruments: An Overview. Oxford Textbook of Public Health.

[B52] World Bank World Development Report 1993: Investing in Health.

[B53] United Nations Development Program (UNDP) (2003). Human Development Report 2003.

[B54] WHO (2005). Health and the Millennium Development Goals Geneva, WHO.

[B55] WHO Constitution of the World Health Organisation New York.

[B56] Arai-Takahashi Y, Martin R, Johnson L (2001). The Role of International Health Law and the WHO in the Regulation of Public Health. Law and the Public Dimension of Health.

[B57] Fidler D, Smith R, Beaglehole R, Woodward D, Drager N (2003). International Law. Global Public Goods for Health: Health Economic and Public Health Perspectives.

[B58] Shibuya K, Ciecierski C, Guindon E, Bettcher D, Evans D, Murray C (2003). WHO Framework Convention on Tobacco Control: Development of an Evidence Based Global Public Health Treaty. British Medical Journal.

[B59] WHO Commission on Macroeconomics and Health (2001). Macroeconomics and Health: Investing in Health for Economic Development, Report of the Commission on Macroeconomics and Health Geneva, WHO.

[B60] Gwatkin D, Guillot M (2000). The Burden of Disease Among the Global Poor: Current Situation, Future Trends, and Implications for Strategy Human Development Network, Health, Nutrition and Population Division, Washington DC, World Bank.

[B61] Anderson G, Chu E (2007). Expanding Priorities – Confronting Chronic Disease in Countries with Low Income. New England Journal of Medicine.

[B62] Marquez P (2005). Addressing Premature Morality and Ill Health Due to Non-Communicable Diseases and Injuries in the Russian Federation.

[B63] Rechel B, Shapo L, McKee M (2004). Millennium Development Goals for Health in Europe and Central Asia: Relevance and Policy Implications World Bank Working Paper No 33 Washington DC, World Bank.

[B64] Waxman A (2004). Why a Global Strategy on Diet, Physical Activity and Health? The Growing Burden of Non-Communicable Diseases. Public Health Nutrition.

[B65] WHO Global Strategy for the Prevention and Control of Noncommunicable Diseases: Report by the Director-General A53/14 (22 March 2000).

[B66] WHO Prevention and Control of Noncommunicable Diseases WHA5317 (20 May 2000).

[B67] WHO Regional Office for Europe European Charter on Counteracting Obesity EUR/06/5062700/8 (16 November 2006) Adopted by EU Ministers at the WHO European Ministerial Conference on Counteracting Obesity: Diet and Physical Activity for Health, Istanbul, Turkey, 15–17 November 2006.

[B68] World Bank World Bank Strategy for Health, Nutrition and Population Results Background Note for a Briefing to the Committee on Development Effectiveness on the Preparation of the New Bank HNP [Health, Nutrition, and Population] Strategy Washington DC, World Bank.

[B69] Prah Ruger J (2005). The Changing Role of the World Bank in Global Health. American Journal of Public Health.

[B70] Prah Ruger J (2005). What Will the New World Bank Head Do for Global Health?. Lancet.

[B71] Williamson J (2000). What Should the World Bank Think of the Washington Consensus?. The World Bank Research Observer.

[B72] Massey D, Sanchez M, Behrman J (2006). Of Myths and Markets. Annals, AAPSS.

[B73] World Bank (2005). Economic Growth in the 1990s: Learning from a Decade of Reform Washington DC, The World Bank.

[B74] World Bank/International Monetary Fund (2005). 2005 Review of the PRS Approach: Balancing Accountabilities and Scaling Up Results Washington DC, The World Bank/IMF.

[B75] Roberts J (2005). Millennium Development Goals: Are International Targets Now More Credible?. Journal of International Development.

[B76] World Bank (1997). Health, Nutrition, and Population Sector Strategy Paper Washington DC, The World Bank.

[B77] World Bank, Human Development Network (2002). Public Health and World Bank Operations Health, Nutrition, and Population Services Washington DC, The World Bank.

[B78] Ebrahim S, Smeeth L (2005). Non-communicable Diseases in Low and Middle-Income Countries: A Priority or a Distraction?. International Journal of Epidemiology.

[B79] Garrett L (2007). The Challenge of Global Health. Foreign Affairs.

[B80] United Nations, General Assembly Road Map Towards the Implementation of the United Nations Millennium Declaration: Report of the Secretary-General. UN Doc A/56/326 (6 September 2001).

[B81] United Nations, General Assembly Follow-up to the Outcome of the Millennium Summit UN Doc A/RES/55/162 (18 December 2000).

[B82] Wagstaff A, Claeson M, Hecht R, Gottret P, Fang Q, Jamison D et al (2006). Millennium Development Goals for Health: What Will it Take to Accelerate Progress?. Disease Control Priorities in Developing Countries.

[B83] World Bank HNP Millennium Development Goals. http://web.worldbank.org/WBSITE/EXTERNAL/TOPICS/EXTHEALTHNUTRITIONANDPOPULATION/EXTHNPMDGS/0,,menuPK:64207167~pagePK:64207177~piPK:64207192~theSitePK:563129,00.html.

[B84] Taylor A, Bettcher D, Peck R, Smith R, Beaglehole R, Woodward D, Drager N (2003). International Law and the International Legislative Process: the WHO Framework Convention on Tobacco Control. Global Public Goods for Health: Health Economic and Public Health Perspectives.

[B85] Taylor A (1996). An International Regulatory Strategy for Global Tobacco Control. Yale Journal of International Law.

[B86] Joossens L (2000). From Public Health to International Law: Possible Protocols for Inclusion in the Framework Convention on Tobacco Control. Bulletin of the World Health Organization.

[B87] Hammond R, Assunta M (2003). The Framework Convention on Tobacco Control: Promising Start, Uncertain Future. Tobacco Control.

[B88] Daynard R (2003). Lessons from Tobacco Control for the Obesity Control Movement. Journal of Public Health Policy.

[B89] Bettcher D, Shapiro I (2001). Tobacco Control in an Era of Trade Liberalisation. Tobacco Control.

[B90] Smith R (2006). Trade and Public Health: Facing the Challenges of Globalisation. Journal of Epidemiology and Community Health.

[B91] Labonte R, Sanger M (2006). Glossary of the World Trade Organisation and Public Health. Journal of Epidemiology and Community Health.

[B92] Correa C (2000). Implementing National Public Health Policies in the Framework of WTO Agreements. Journal of World Trade.

[B93] WHO/WTO (2002). WTO Agreements and Public Health: A Joint Study by the WHO and the WTO Secretariat Geneva, WTO/WHO.

[B94] European Communities – Measures Affecting the Approval and Marketing of Biotech Products (WT/DS291, WT/DS292, WT/DS293).

[B95] Cardwell D Bloomberg Donating $125 Million to Anti-Smoking Efforts. New York Times.

[B96] WTO, General Agreement on Tariffs and Trade 1994 (GATT).

[B97] WTO, Agreement on the Application of Sanitary and Phytosanitary Measures (SPS Agreement).

[B98] WTO, Agreement on Technical Barriers to Trade (TBT Agreement).

[B99] Kowal P, Lopez A (2003). Child Survival. Lancet.

[B100] Esson K, Leeder S (2004). The Millennium Development Goals and Tobacco Control: An Opportunity for Global Partnership.

[B101] Fuster V, Voûte J (2005). MDGs: Chronic Diseases are not on the Agenda. Lancet.

[B102] Haines A, Cassels A (2004). Can the Millennium Development Goals be Attained?. British Medical Journal.

[B103] Evans D, Adam T, Tan-Torres Edejer T, Lim S, Cassels A, Evans T (2005). Achieving the Millennium Development Goals for Health: Time to Reassess Strategies for Improving Health in Developing Countries. British Medical Journal.

[B104] Spinaci S, Currat L, Shetty P, Crowell V, Kehler J (2006). Tough Choices: Investing in Health for Development: Experiences from National Follow-up to the Commission on Macroeconomics and Health Geneva, WHO.

[B105] WHO/FAO (2005). Fruit and Vegetables for Health Report of a Joint FAO/WHO Workshop, 1–3 September 2005 Kobe Japan, WHO/FAO.

[B106] WHO International Code of Marketing of Breast-Milk Substitutes WHA3422 (21 May 1981).

[B107] Codex Alimentarius Commission, Joint FAO/WHO Food Standards Programme, Discussion Paper Prepared by WHO in Cooperation with FAO: Implementation of the WHO Global Strategy on Diet, Physical Activity and Health: Action that Could be Taken by Codex Twenty-eighth Session, Rome, 4–9 July 2005, CAC/28 LIM/6.

[B108] Codex Alimentarius Commission, Joint FAO/WHO Food Standards Programme, Codex Committee on Nutrition and Foods for Special Dietary Uses Discussion Paper Prepared by WHO in Cooperation with FAO: Implementation of the WHO Global Strategy on Diet, Physical Activity and Health: Development of "Actions Document" for Codex Twenty-seventh Session, Bonn, Germany, 21–25 November 2005, CX/NFSDU 05/27/2-Add1.

[B109] Codex Alimentarius Commission, Twenty-ninth Session, Geneva, Switzerland, 3–7 July 2006. Report, ALINORM 06/29/41.

[B110] Braithwaite J, Drahos P (2000). Global Business Regulation.

[B111] Sikes L (1998). FDA's Consideration of Codex Alimentarius Standards in Light of International Trade Agreements. Food and Drug Law Journal.

[B112] WHO, Intergovernmental Working Group on Revision of the International Health Regulations Review and Approval of Proposed Amendments to the International Health Regulations: Relations with Other International Instruments A/IHR/IGWG/INFDOC/1 (30 September 2004).

[B113] World Bank (1999). Curbing the Epidemic: Governments and the Economics of Tobacco Control.

[B114] World Bank (2006). Repositioning Nutrition as Central to Development: A Strategy for Large-Scale Action.

[B115] Brunner E, Cohen D, Toon L (2001). Cost Effectiveness of Cardiovascular Disease Prevention Strategies: A Perspective on EU Food Based Dietary Guidelines. Public Health Nutrition.

[B116] Raymond S, Greenberg H, Liu H, Leeder S (2004). Civil Society Confronts the Challenge of Chronic Illness. Development.

[B117] Chopra M, Galbraith S, Darnton-Hill I (2002). A Global Response to a Global Problem: the Epidemic of Overnutrition. Bulletin of the World Health Organization.

[B118] Brownell K, Nestle M The Sweet and Lowdown on Sugar. The New York Times.

[B119] Lang T, Rayner G, Kaelin E The Food Industry, Diet, Physical Activity and Health: A Review of Reported Commitments and Practice of 25 of the World's Largest Food Companies April 2006.

[B120] European Commission, Health and Consumer Protection Directorate-General Physical Activity and Health – EU Platform for Action.

[B121] EU Platform on Diet, Physical Activity and Health.

[B122] EU Platform on Diet, Physical Activity and Health: Synopsis of Commitments, Annual Report 2007 (13 March 2007).

[B123] European Union, Delegation of the European Commission to the USA EU Platform on Diet, Physical Activity and Health: European Commission Commends Companies for their Commitments to Fighting Obesity No 96/06.

[B124] Alliance for a Healthier Generation School Beverage Guidelines.

[B125] Alliance for a Healthier Generation Competitive Food Guidelines.

[B126] Clinton Foundation, Press Release Alliance for a Healthier Generation – Clinton Foundation and American Heart Association – and Industry Leaders Set Healthy School Beverage Guidelines for U.S.Schools. http://www.healthiergeneration.org/media.aspx.

[B127] Clinton Foundation, Press Release President Clinton and American Heart Association Announce Joint Agreement Between Alliance for a Healthier Generation and Food Industry Leaders to Set Healthy Standards for Snacking in School. http://www.healthiergeneration.org/media.aspx.

[B128] Caraher M, Cowburn G (2005). Taxing Food: Implications for Public Health Nutrition. Public Health Nutrition.

[B129] Beaglehole R, Yach D (2003). Globalisation and the Prevention and Control of Non-Communicable Disease: the Neglected Chronic Diseases of Adults. Lancet.

[B130] Yach D, Hawkes C, Epping-Jordan J, Galbraith S (2003). The World Health Organization's Framework Convention on Tobacco Control: Implications for Global Epidemics of Food-related Deaths and Disease. Journal of Public Health Policy.

[B131] Chopra M, Darnton-Hill I (2004). Tobacco and Obesity Epidemics: Not So Different After All?. British Medical Journal.

[B132] Lee E (2005). The World Health Organization's Global Strategy on Diet, Physical Activity, and Health: Turning Strategy into Action. Food and Drug Law Journal.

[B133] Evans M, Sinclair R, Fusimalohi C, Liava'a V (2001). Globalization, Diet, and Health: An Example from Tonga. Bulletin of the World Health Organization.

[B134] Redmond P (2003). Transnational Enterprise and Human Rights: Options for Stand Setting and Compliance. International Lawyer.

[B135] Lewin A, Lindstrom L, Nestle M (2006). Food Industry Promises to Address Childhood Obesity: Preliminary Evaluation. Journal of Public Health Policy.

[B136] Jack W (1999). Principles of Health Economics for Developing Countries World Bank Institute Development Studies Washington DC, The World Bank.

[B137] Ezzati M, Vander Hoorn S, Lopez A, Danaei G, Rodgers A, Lopez A, Mathers C, Ezzati M, Jamison D, Murray C (2006). Comparative Quantification of Mortality and Burden of Disease Attributable to Selected Risk Factors. Global Burden of Disease and Risk Factors.

[B138] WHO/FAO (2006). Marketing of Food and Non-Alcoholic Beverages to Children Report of a WHO Forum and Technilcal Meeting, Oslo, Norway, 2–5 May 2006 Geneva, WHO.

[B139] Lawlor D, Chaturvedi N (2006). Treatment and Prevention of Obesity – Are There Critical Periods for Intervention?. International Journal of Epidemiology.

[B140] WHO (2006). Global Strategy on Diet, Physical Activity and Health: A Framework to Monitor and Evaluate Implementation Geneva, WHO.

[B141] AP Denmark Leads the Way in Banning Killer Fat. International Herald Tribune.

[B142] Lueck T, Severson K New York Bans Most Trans Fats in Restaurants. New York Times.

[B143] New York City, Department of Health and Mental Hygiene, Board of Health Notice of Adoption of an Amendment (§81.08) to Article 81 of the New York City Health Code. http://www.nyc.gov/html/doh/html/notice/notice.shtml.

[B144] New York City, Department of Health and Mental Hygiene, Press Release, Board of Health Notice of Adoption of an Amendment (§81.50) to Article 81 of the New York City Health Code. http://www.nyc.gov/html/doh/html/notice/notice.shtml.

[B145] Food Safety Authority of Ireland Salt and Health. http://www.fsai.ie/industry/salt/salt2.asp.

[B146] Warner M The War Over Salt. The New YorkTimes.

[B147] Appel L (2006). Salt Reduction in the United States. British Medical Journal.

[B148] Havas S, Roccella E, Lenfant C (2004). Reducing the Public Health Burden from Elevated Blood Pressure Levels in the United States by Lowering Intake of Dietary Sodium. American Journal of Public Health.

[B149] Ofcom (Office of Communications, UK) Television Advertising of Food and Drink Products to Children: Statement and Further Consultation. http://www.ofcom.org.uk/consult/condocs/foodads_new/foodads3.pdf.

[B150] Ofcom (Office of Communications, UK) Television Advertising of Food and Drink Products to Children. http://www.ofcom.org.uk/consult/condocs/foodads_new/statement/.

[B151] Mello M, Studdert D, Brennan T (2006). Obesity – The New Frontier of Public Health Law. New England Journal of Medicine.

[B152] Gostin L (2007). Law as a Tool to Facilitate Healthier Lifestyles and Prevent Obesity. Journal of the American Medical Association.

[B153] 42 USC §1751ff, §1771ff.

[B154] (2006). Senate Eyes School Junk Food. JAMA.

[B155] Child Nutrition Promotion and School Lunch Protection Act of 2006 (S2592 IS), Bill introduced into the United States Senate.

